# Reassembling haplotypes in a mixture of pooled amplicons when the relative concentrations are known: A proof-of-concept study on the efficient design of next-generation sequencing strategies

**DOI:** 10.1371/journal.pone.0195090

**Published:** 2018-04-05

**Authors:** Louis Ranjard, Thomas K. F. Wong, Allen G. Rodrigo

**Affiliations:** The Research School of Biology, The Australian National University, Australia; Oklahoma State University, UNITED STATES

## Abstract

Next-generation sequencing can be costly and labour intensive. Usually, the sequencing cost per sample is reduced by pooling amplified DNA = amplicons) derived from different individuals on the same sequencing lane. Barcodes unique to each amplicon permit short-read sequences to be assigned appropriately. However, the cost of the library preparation increases with the number of barcodes used. We propose an alternative to barcoding: by using different known proportions of individually-derived amplicons in a pooled sample, each is characterised *a priori* by an expected depth of coverage. We have developed a Hidden Markov Model that uses these expected proportions to reconstruct the input sequences. We apply this method to pools of mitochondrial DNA amplicons extracted from kangaroo meat, genus *Macropus*. Our experiments indicate that the sequence coverage can be efficiently used to index the short-reads and that we can reassemble the input haplotypes when secondary factors impacting the coverage are controlled. We therefore demonstrate that, by combining our approach with standard barcoding, the cost of the library preparation is reduced to a third.

## Introduction

Next-generation sequencing technologies that utilise many short fragments of DNA now allow scientists to assemble the sequences of individual genes and genomes. Although, full genome sequencing approaches are becoming more feasible, targeted sequencing methods such as amplicon sequencing are still used as a cost-effective means of genotyping large numbers of individuals [1–5].

A standard approach to short-read sequencing is to pool amplicons from multiple individuals into a single sample that is subsequently sequenced. To identify the provenance of short-reads, this method requires a unique DNA sequence tag, or “barcode”, to be attached to the short fragments of DNA obtained from the amplicon(s) of each individual. The barcodes allow the sequences associated with a particular individual to be separated computationally. This approach requires a separate library preparation for each individual, thus increasing this portion of the sequencing cost linearly as more individuals are added to the sample. In fact, costs associated with library preparation represent a large component of the total cost of DNA sequencing experiments (~20%, [6]).

Because the cost of the experiment increases with the number of individuals, pooling strategies have been developed [6] to reduce the total number of sequencing samples to be barcoded. These allow researchers to increase the number of individuals sequenced [7] by grouping multiple individuals under the same barcode or grouping all individuals together without using barcodes at all. Although the best estimate of population parameters is achieved using the sequences of each individual [8], pooling approaches can offer a way to estimate some parameters [9] using a larger population sample size at reduced cost. However, in most pooled experiments, the sequences of each individual remain unknown. Thus, certain types of analyses, e.g. phylogenetic and genealogy-based methods, cannot be performed.

Here, we investigate an approach that allows us to pool the individuals under study while still recovering the unique sequence of each individual. Barcode-free sequencing of pooled individuals has been proposed previously; [10] focused on distantly related individuals (e.g. single representatives of different species, genera, or higher taxonomic groups) that have diverged sufficiently so that they can be separated based on available reference sequences. For these methods to work, there needs to be a high likelihood of seeing individual-specific nucleotide differences in each short read, so that the reads can be matched to the reference sequences easily.

But what if the variants are from the same or closely-related species? In this paper, we propose a method that allows us to reconstruct the sequence(s) associated with each individual without the need for a unique barcode. Instead, we use known relative concentrations of input DNA as frequency “markers” associated with the sequences from each individual. By aligning the short reads to a reference sequence, the nucleotide sequence for each individual at each position can be inferred from the expected coverage induced by the known concentrations.

Our assumption is that the short read coverage of the original DNA sequences is proportional to the amount of DNA loaded on the sequencing run. Variation in the read coverage of Illumina sequencing technology is known to be sensitive to GC content [11–12] as well as to specific motifs [13–14]. We expect coverage of the entire mixture to vary along the length of the amplified fragments. We further assume, therefore, that the variation in coverage is not systematically biased in favour or against a subset of amplicons in the mixture, and therefore, at any given site, the expected proportion of coverage for each amplicon in the mixture remains the same. It is therefore critical to correctly model coverage variation and particular care is given to this matter in our approach.

As a proof-of-concept study, we investigate the pooling of three haplotypes in known relative concentrations. Such a design potentially divides the cost of the library preparation by three; we discuss the cost of this strategy against other commonly applied sequencing strategies below. We sequence three non-overlapping mitochondrial regions from each of three independent kangaroo samples, genus *Macropus*. In our design, the haplotype sequence from each individual has a different known contribution in the pools. We use a Hidden Markov Model (HMM) where the hidden states are the three individuals from which the amplified sequences have been obtained. The aim is to reconstruct the amplified fragments from the mitochondrial DNA of each of the three individual kangaroos.

## Materials and methods

The following terminology will be used in this paper: a haplotype refers to the genetic sequence of a single individual from which an amplicon is derived. An amplicon is a single amplified product originating from a single haplotype. Samples refer to sequencing samples and can therefore be made of a single amplicon or a mixture of amplicons.

### DNA material collection and sequencing

In Australia, kangaroos are not farmed. Instead, wild kangaroos are culled by licensed hunters, and these are sold to meat processors and butchers. Kangaroo steaks (open range kangaroo steak, primary barcodes 2802693006493, 2802671007078 and 2802693008831, Macromeat, 51–52 Lavinia Street, Athol Park SA) were purchased at local supermarkets in the Australian Capital Territory (Coles Supermarkets Australia Pty Ltd and Woolworths Ltd) on three separate occasions over several weeks (2016 September 28^th^, October 9^th^ and November 1^st^). We did this to avoid sampling the same animal because the exact provenances were unknown. The kangaroo species were also unknown at the time of purchase but statistics from the Department of the Environment and Energy of Australia indicate that they must be of genus *Macropus*, and most likely Eastern grey kangaroo, *Macropus giganteus*, as it is the largest population with highest quota in New South Wales [15]. The three kangaroo individuals are referred as KA, KB and KC hereafter.

### DNA extraction

Approximately 30 mg of meat was excised and homogenized for each individual. Genomic DNA was extracted using Thermo Scientific GeneJET Genomic DNA Purification Kit (silica-based membrane spin column) following manufacturer protocol. For each individual, approximately 200 μl of high molecular weight DNA was obtained at around 5 ng/μL concentration as measured on a Qubit fluorometer (Invitrogen, Grand Island, NY).

### Amplification

**Fig 1:**
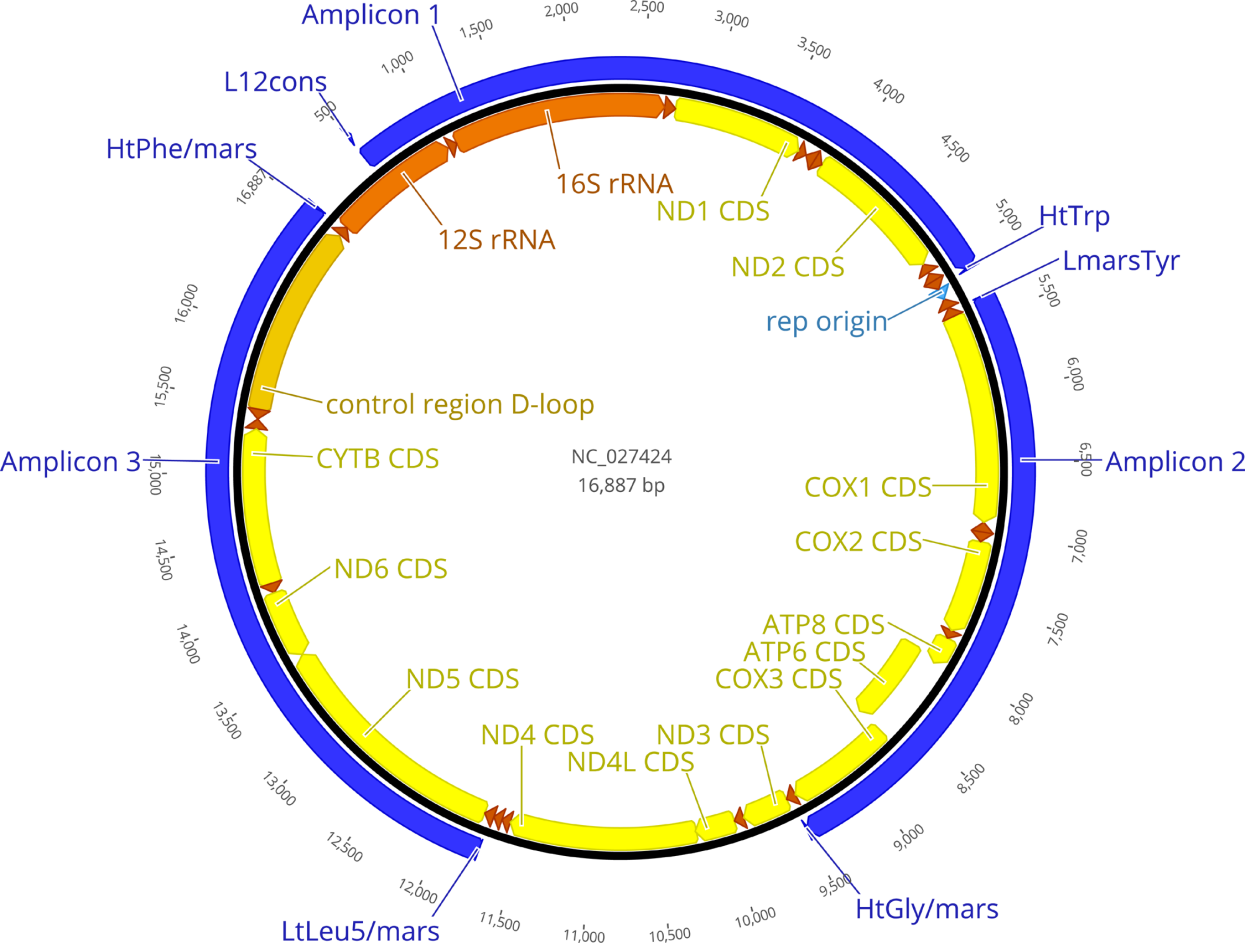
Three mitochondrial amplicons with predicted genomic location on the eastern grey kangaroo reference sequence. The locations are inferred from the position of the three pairs of PCR primers from [16].

Three mitochondrial pairs of primers (Fig 1 and Table 1) were selected from [16] so that three non-overlapping mitochondrial amplicons could be generated, covering ~83% of the haploid genome. Predicted amplicon lengths according to the eastern grey kangaroo, *Macropus giganteus*, reference mitochondrial genome ([17]; Genbank accession number NC_027424) were 4640, 4151 and 5194 nucleotides. Fragments were amplified using Takara PrimeSTAR GXL DNA Polymerase following manufacturer protocol for 3-step PCR (30 cycles, each being: 98°C for 10 sec., 55°C for 15 sec., 68°C for 5 min.). The second amplicon had a lower annealing temperature and therefore an annealing temperature of 52°C was used in the second step to ensure amplification. Concentration of products after PCR clean-up was measured on a Qubit fluorometer at [19.9–90.4] ng/μL.

**Table 1:** Primer sequences used for amplification [16].

Primer name		Gene	Primer sequence 5’-3’	Position in NC_027424 sequence
L12cons (76)	Forward	12S	GGGATTAGATACCCCACTAT	500–519
HtTrp (637)	Reverse	tRNA-Trp	GCTTAAGGCTTTGAAGGC	5123–5140
HtTrp (637)	Forward	tRNA-Tyr	GTCTTTGAATTTACAGTT	5345–5362
HtGly/mars (641)	Reverse	tRNA-Gly	GAACTTAATGATTGGAAA	9479–9496
LtLeu5/mars (257)	Forward	tRNA-Leu	ATCCRTTGGTCTTAGGAA	11749–11766
HtPhe/mars (382)	Reverse	tRNA-Phe	CCATCTAAGCATTTTCAGT	29–47

### Pooled samples preparation

A total of 10 pooled samples were prepared by mixing the amplicons in relative concentrations of 1:2:5 following DNA quantification to prevent ambiguity in the expected coverage. This ratio–which translates into the relative frequencies 0.125, 0.25 and 0.625 –was chosen to generate large differences in expected frequencies between all possible subsets of variant assignments (Table 2).

**Table 2:** Expected proportions for the haplotypes (H1, H2 and H3) regions when some are identical. Identical haplotypes are grouped in brackets. Note that the expected proportions for the region where some haplotypes are identical (in bold) are all different and distinct from the proportions of the individual haplotypes.

**Expected observed read coverage proportions**	0.125	**0.375**	0.25	0.125	**1**
	0.25			**0.875**	
			**0.75**		
	0.625	0.625			
**Haplotype grouping by identity**	H1,H2,H3	(**H1+H2**),H3	H2,(**H1+H3**)	H1,(**H2+H3**)	(**H1+H2+H3**)

### Sequencing

Library preparation and sequencing were performed by the Biomolecular Resource Facility at the Australian National University. Amplified fragments were sequenced both individually, i.e. single-amplicon samples, and as mixtures of known proportions, i.e. pooled samples. Technical replicates were performed in order to assess the robustness and reproducibility of the haplotype reconstruction approach and ensure the correct assembly of the reference sequences for each individual amplicon (Table 3). Illumina Nextera paired-end libraries were constructed and sequencing was performed on an Illumina MiSeq machine with read length of 2 x 151 bp. Each of the 9 amplicons were sequenced twice in two barcoded single-amplicon sequencing samples, except for one that was sequenced only once (KB—amplicon 3). In total, 28 samples were sent for sequencing: 10 pooled and 18 single-amplicon samples.

**Table 3:** Proportions of the different individuals in the pooled experiments for each amplicon (Amp.). Because of limitation on the amount of sequencing data we could generate, we could only focus on replicating amplicon 1 pools.

Individual KA			Individual KB			Individual KC			Number of reads	Coverage (1|2|5)	Data ID
**Amp. 1**	**Amp. 2**	**Amp. 3**	**Amp. 1**	**Amp. 2**	**Amp. 3**	**Amp. 1**	**Amp. 2**	**Amp. 3**			
0.125	-	-	0.25	-	-	0.625	-	-	498,832	2,000x | 4,100x | 10,100x	10
-	0.125	-	-	0.625	-	-	0.25	-	526,877	2,400x | 4,800x | 12,000x	11
-	-	0.25	-	-	0.625	-	-	0.125	594,381	2,200x | 4,400x | 11,000x	12
0.25	-	-	0.625	-	-	0.125	-	-	398,916	1,600x | 3,200x | 8,100x	23
-	0.125	-	-	0.25	-	-	0.625	-	350,216	1,600x | 3,200x | 8,000x	24
-	-	0.625	-	-	0.125	-	-	0.25	247,402	900x | 1,800x | 4,500x	25
0.625	-	-	0.125	-	-	0.25	-	-	419,923	1,700x | 3,400x | 8,500x	27
0.125	-	-	0.625	-	-	0.25	-	-	164,783	700x | 1,300x | 3,400x	28
0.625	-	-	0.25	-	-	0.125	-	-	355,690	1,400x | 2,900x | 7,200x	29
0.25	-	-	0.125	-	-	0.625	-	-	260,027	1,000x | 2,100x | 5,300x	30

### Haplotype reconstruction

The assembly of the haplotypes began by mapping the reads to the reference sequence of the type species for the genus *Macropus* (eastern grey kangaroo mitochondrial genome, Genbank NC_027424) in Geneious version 10.2.3 (http://www.geneious.com, [18]). Single-amplicon samples with high coverage were assembled against this reference, and subsequently, were taken as the true haplotypes for each of the three individuals. These true haplotype sequences were then used to assess the reconstructed haplotypes obtained from the mixed samples. Mismatches and indels in the alignment of each reconstructed haplotype from the mixed samples and the equivalent haplotype recovered from single-amplicon sequencing were counted as errors.

For the single-amplicon samples, pre-processing and error correction of the short-read sequences proceeded as follows. Illumina Nextera adapters were removed from the short read sequences and the reads were trimmed with a quality threshold of Phred score 6 using the program BBDuk 37.25 (Brian Bushnell within Geneious 10.2.3). Short reads less than 10 bp long were discarded. Paired-end reads were error corrected and kmer normalized using the program BBNorm 37.25 (Brian Bushnell within Geneious 10.2.3). The kmer target coverage level was set to 50 and minimum kmer depth to 100 because of the high coverage available. The paired-end reads were mapped to the reference (NC_027424) using the Geneious built-in mapper and the consensus base called at each position using “highest quality threshold” option. Briefly, the total quality is summed for each potential base call, and a specific base is called if it exceeds 60% of the total quality. Finally, both 5’ and 3’ ends of the newly assembled amplicons were trimmed using a Phred quality score threshold of 40, resulting in the trimming of about 20 nucleotides at each end. No ambiguous bases were obtained after this procedure was applied.

For the mixed samples, pre-processing of the short reads consisted in trimming the low quality bases, a maximum of 5 bases below Phred score 20 was allowed per read, and discarding the reads with a mean quality Phred score below 20 [19]. Illumina Nextera adapters were then removed using the program BBDuk 36.86 (Brian Bushnell).

The reconstruction of each haplotype from the mixed samples was performed in three steps. First, the short reads were mapped to the reference sequence. Second, a multinomial model parametrised with the expected known proportion of each haplotype (Table 2) was applied to the variable sites in the alignment to identify the regions in the alignment that differ for all three haplotypes. Finally, starting at the variable regions with the highest likelihood for the multinomial model with three haplotypes (S1 Appendix), a Hidden Markov Model was applied to identify the nucleotide of each haplotype at each position. The HMM was run in both directions from these starting sites. The following sections describe each of these in detail.

### Mapping to a reference sequence

The program Bowtie 2 (version 2.2.6, [20]) was used to map the reads from the pooled samples to the reference sequence (Genbank NC_027424). The corresponding SAM file was converted to a multiple sequence alignment using a custom C++ program to insert gaps to the mapping position of the reads. This step is necessary because when a specific read requires a gap to be aligned to the reference, this gap needs to be added into the alignment of all the other reads to obtain consistent alignment positions. The resulting new SAM file was analysed to reconstruct the haplotypes of the different pooled samples.

### Error rate estimation

To obtain an “error rate” for the single-amplicon samples, various numbers of reads were sub-sampled to simulate 10 different coverage, from 10x to 1000x. The reads were mapped to the eastern-grey reference sequence using Bowtie 2 mapper (version 2.2.6, [20]) and Freebayes variant caller (version 1.1.0-46-g8d2b3a0, [21]) was used to identify varying positions between the reads and the reference. Then, the consensus sequences were reconstructed with BCFtools (version 1.6, http://samtools.github.io/bcftools). The total number of mismatches (including insertions or deletions) between the reconstructed sequence and the true sequences was divided by the lengths of the true haplotypes to obtain the error rate. For each coverage, a total of 10 sub-sampled datasets were generated. The error rate for the pooled samples was calculated in a similar manner.

### Dirichlet-multinomial hidden Markov Model

We consider the sequencing of three haplotypes pooled together in known proportions (1:2:5). The coverage of the reads throughout the mapping of the sequenced reads onto the reference sequence (*Macropus giganteus*, Genbank NC_027424) is modelled according to a Dirichlet-multinomial distribution [22–23] with three categories corresponding to the three haplotypes in the mixture. To smooth the coverage across the alignment, we used a sliding window of size 50 in our analysis. At each window, the set of unique sub-sequences and their coverage is first extracted from the alignment. The sub-sequences are ordered according to their coverage and no more than the three most frequent sub-sequences are retained (see S1 Appendix for details),

As noted above, at any given site along the alignment of short-reads against the reference, nucleotide frequencies can be modelled by the expected frequencies induced by the input concentrations of the three haplotypes. The same is true for the frequencies of the sub-sequences in each window. If all three haplotypes have a different sub-sequence in a given window, we expect that the coverage will be some multinomial sample from [0.125, 0.25, 0.625], the ratios of the input concentrations. However, it is possible that two haplotypes share a common sub-sequence that is not shared by the third. In these cases, the expected coverage will be equal to the sum of the concentrations of the two haplotypes that share that sub-sequence. In all, there are four possible assortments of variable sub-sequences for each window, corresponding to the four different ways that haplotypes may share (or not share) common sub-sequences (Table 2).

We begin the HMM by finding a window of 50 nucleotides for which there are three different sub-sequences, where the joint probability of their respective coverages given an expected ratio of [0.125, 0.25, 0.625] is maximised. This window is selected as the starting point of the HMM, with the HMM running in both directions. Briefly, the HMM is applied as follows (see S1 Appendix for a formal definition):

- for each position in the alignment, the observations consist in the coverage of the three most frequent sub-sequences.
- 27 states correspond to the assignment of each local subsequence to each haplotype. For example in state 1, all the three haplotypes share the same subsequence 1, the most frequent, and it is assumed that the other sub-sequences result from sequencing or mapping errors,
- transition probabilities are calculated from the sum of the Hamming distances between the sub-sequences assigned to each haplotype in two consecutive analysis windows,
- emission probabilities are defined according to a Dirichlet multinomial. The parameters of this distribution are estimated by considering the local average coverage of each haplotypes according to the last windows. Typically, regional coverage is estimated from the 400 previous nucleotide positions using a triangular analysis window. This distribution models the probability of observing the coverage under each state.

In each analysis window and for each state, the previous state with the highest probability is recorded along with the corresponding likelihood of the path. The three haplotypes are finally reconstructed by tracing back the path with the highest likelihood using the Viterbi algorithm [24], from the HMM run in each direction from the starting point. The two resulting sequences are then joined to assemble the full length haplotype.

### Assessing the effect of the overall short read coverage

When coverage is low, it is expected that random technical errors may potentially disrupt the targeted proportions. Subsets of sequenced short reads were randomly sub-sampled in the pooled samples to simulate various coverage values. The size of these subsets was chosen so that the individual in the lowest proportion (12.5%) was covered at 10x, 30x, 50x, 100x, 200x with respectively 2 and 5 times as much coverage for the two other haplotypes (Table 4). For each of these 5 coverage values and for each of the 10 pooled samples, 10 subsets were sub-sampled so that, in total, 500 new datasets were generated. The Hidden Markov Model was then applied to reconstruct the three pooled haplotypes individually.

**Table 4:** Number of reads sampled for building the subsets. For simplicity, it is assumed that the amplicon length is 5,000bp.

Target coverages in pooled samples	Number of sampled reads
10x | 20x | 50x	1,333
30x | 60x | 150x	4,000
50x | 100x | 250x	6,667
100x | 200x | 500x	13,333
200x | 400x | 1000x	26,667

## Results

### Single-amplicon assembly to identify true individual haplotypes

On average, the coverage for the single-amplicon samples was 3360x but variation across amplicons was substantial (Table 5). After trimming and error correction the average read length was 147.9 [10–151]. After kmer normalization the coverage of the single-amplicon samples was 72x [69–78].

**Table 5:** Single-amplicon samples sequencing statistics per amplicon.

		Amplicon 1	Amplicon 2	Amplicon 3
	**Predicted length**	**4641**	**4152**	**5140**
**KA**	Number of read pairs	107293	98121	73976
	coverage	3,490.89	3,568.47	2,173.22
	Assembly length	4619	4134	5129
**KB**	Number of read pairs	110244	91556	96154
	coverage	4,768.65	3,874.20	6,680.97
	Assembly length	4622	4126	5129
**KC**	Number of read pairs	91537	48125	40106
	coverage	2,898.53	1,875.71	906.46
	Assembly length	4623	4126	5132

**Fig 2:**
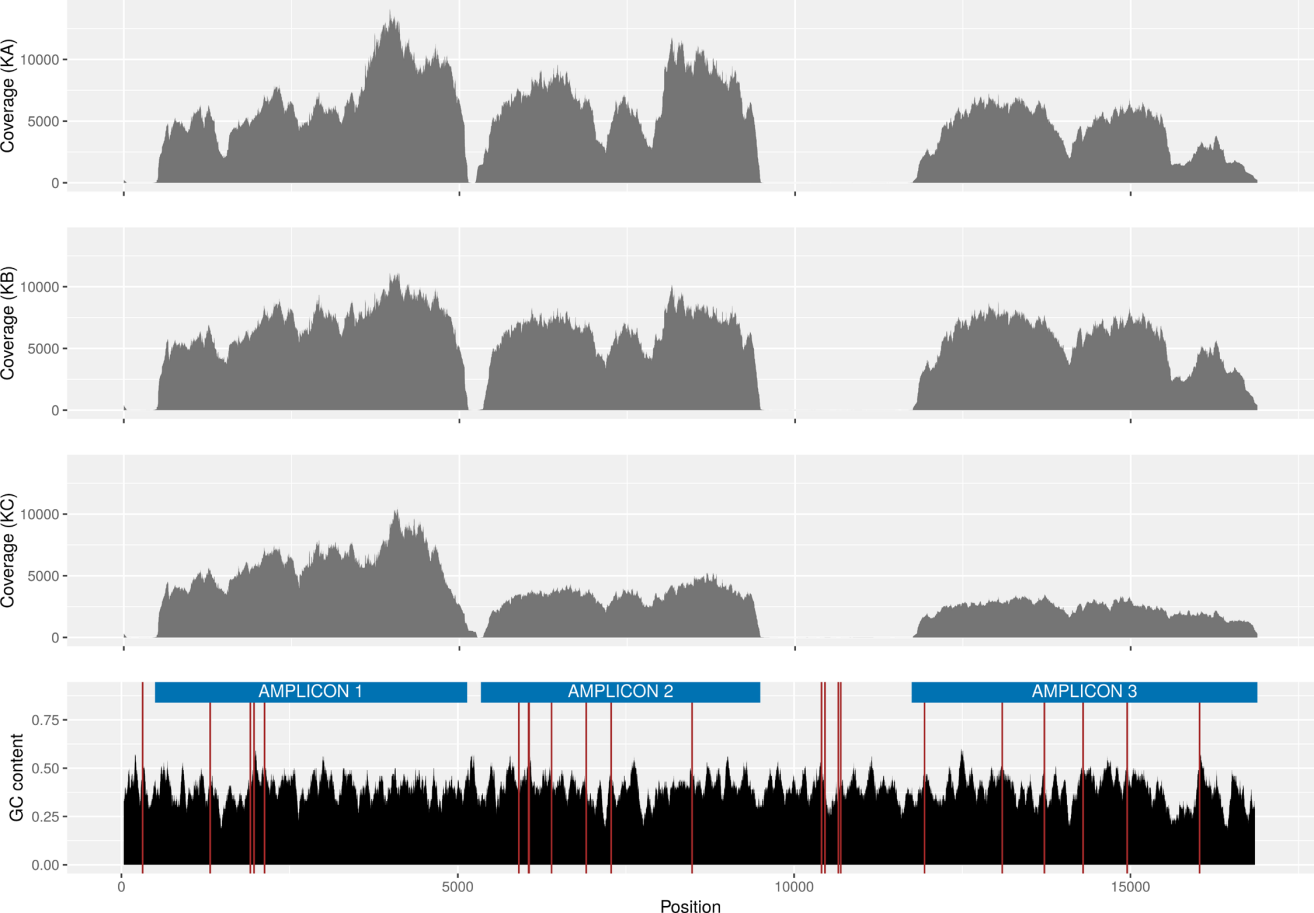
Amplicons sequencing coverage. Sequencing coverage of the three amplicons for the three individuals obtained from the single-amplicon samples. Each amplicon/individual was barcoded separately but are represented here on one graph per individual. The bottom track represents the GC content of the eastern grey kangaroo reference sequence as well as the location of the motifs ‘CCNGCC’ known for affecting Illumina sequencing coverage.

**Fig 3:**
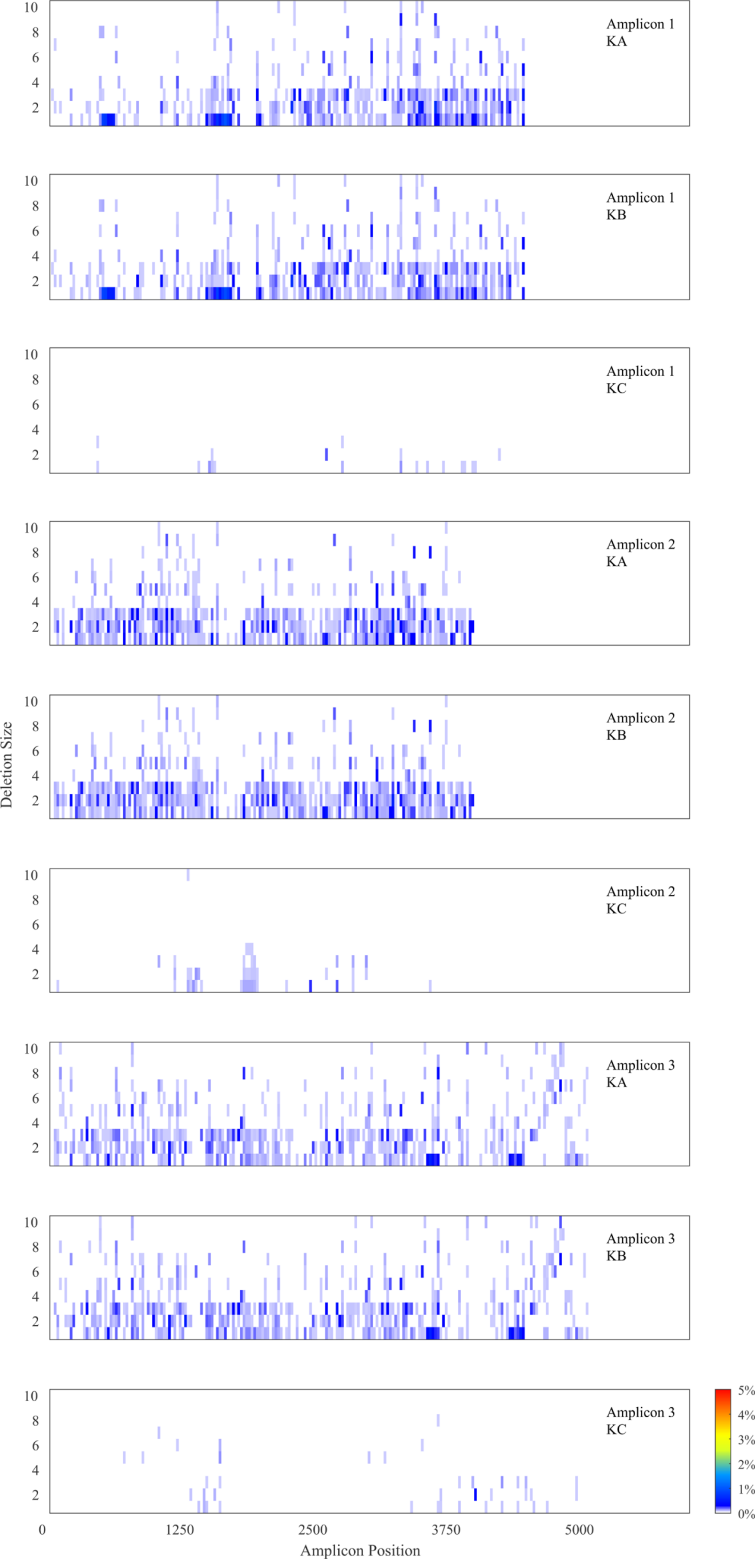
Mapping deletion patterns. Percentage of reads containing deletions when mapped to the Amplicon 3 reference.

The patterns of variation in read coverage of the three amplicons was mostly consistent across individuals (Fig 2) with peaks and troughs around the same mitochondrial genomic regions. Overall, the individual KC presented less variation in coverage, especially for amplicons 2 and 3. Interestingly, the pattern of coverage variation in amplicon 3 for individual KC was different to the two other individuals. In particular, the control region D-loop, located toward the end of amplicon 3, exhibited a peak in coverage for KA and KB which is not visible for KC (Fig 2). Coverage of KA and KB showed higher correlation between each other than with KC (Table 6). Moreover, the mapping of the reads in this region necessitated larger and higher number of deletions for KA and KB reads than for KC. Overall, no major differences in deletion patterns across the individuals KA and KB was observed (Fig 3).

**Table 6:** Correlation coefficient between coverage of every pair of amplicons.

	KA-KB	KA-KC	KB-KC
Amplicon 1	0.9041198	0.6992038	0.8633379
Amplicon 2	0.9570394	0.8307307	0.8574292
Amplicon 3	0.9799699	0.8321273	0.7932484
All	0.9454729	0.8208656	0.8368833

A positive correlation was found between Illumina read coverage and GC content of the eastern grey kangaroo reference sequence (Table 7). The relationship was the strongest for Amplicon 3 with a Pearson correlation coefficients *r* of [0.48, 0.52, 0.41] for respectively KA, KB and KC, all statistically different from 0 with all p-values<<0 (tested with the R *cor*.*test* function, https://www.R-project.org). Amplicons 1 and 2 also showed evidence of a statistically significant positive correlation to a lower extent with [0.09, 0.15, 0.24] and [0.38, 0.37, 0.37] coefficient values respectively.

**Table 7:** Correlation coefficient between coverage and GC content of reference sequence.

	KA	KB	KC
Amplicon 1	0.09437351	0.1539678	0.2411795
Amplicon 2	0.3790031	0.3665274	0.3746213
Amplicon 3	0.4822024	0.5151382	0.4149149

The motif ‘CCNGCC’ is known to potentially cause a steep drop in Illumina read coverage [14] and increase sequencing errors [13]. Mixed results were obtained with the effect of this motif on read coverage of the haplotypes. A positive correlation was measured between the distance to the position of the motif in the sequence and the read coverage for Amplicon 1 while a negative correlation was found for Amplicons 2 and 3, all significantly different from 0 with all p-values≪0 (Table 8).

**Table 8:** Correlation between coverage and distance to the position of a motif ‘CCNGCC’.

	KA	KB	KC
Amplicon 1	0.7640182	0.6018595	0.558282
Amplicon 2	-0.3032632	-0.3508846	-0.3830093
Amplicon 3	-0.2329049	-0.2388449	-0.3831313

The reference sequence for each amplicon and each individual was successfully assembled. Single-amplicon sample sequences were submitted to BLAST for species identification (Table 9). The percentage identity of the alignment was used as a criterion to identify the best matching species since the reference sequence for the mitochondrial genome of the western grey kangaroo, *Macropus fuliginosus*, is only a partial sequence (KJ868120). While individual KC closely matched the eastern grey kangaroo reference (NC_027424) with high identity scores, the individuals KA and KB were more closely related to western grey kangaroo (KJ868120) for all amplicons. Consequently, individuals KA and KB were putatively identified as western grey kangaroo and KC as eastern grey kangaroo.

**Table 9:** Number of nucleotide differences and percentage similarity in the alignment of the reconstructed single-amplicon samples with the western grey partial mitochondrial genome (Genbank KJ868120) and the eastern grey mitochondrial genome (Genbank NC_027424). Because the western grey reference sequence (KJ868120) is only partial, Amplicon 3 could not be fully aligned. Note: Blast hits returned match to LK995454 for eastern grey kangaroo which is 100% similar to NC_0275454 referred through this paper.

	KA		KB		KC	
	**Western-grey (KJ868120)**	**Eastern-grey (NC_027424)**	**Western-grey (KJ868120)**	**Eastern-grey (NC_027424)**	**Western-grey (KJ868120)**	**Eastern-grey (NC_027424)**
**Amplicon 1**	4 (99.9%)	176 (96.2%)	3 (99.9%)	177 (96.2%)	180 (96.1%)	6 (99.9%)
**Amplicon 2**	2 (100%)	216 (94.8%)	2 (100%)	224 (94.9%)	220 (94.7%)	4 (99.9%)
**Amplicon 3**	6 (99.8%)	404 (92.1)	8 (99.8%)	405 (92.1%)	229 (93.8%)	17 (99.7%)

### Pooled samples haplotypes reconstruction

At high coverage, all amplicons were successfully reconstructed for each haplotype by our algorithm. In all experiments, the haplotype with the lowest proportion (12.5%) was the most difficult to reconstruct (Table 10). At low coverage, sequence reconstruction can be hampered by incorrect mapping of the reads. At coverage 60x and above the true western grey kangaroo haplotype is successfully reconstructed using the eastern grey reference sequence (Table 11).

**Table 10:** Error rates for the reconstructed haplotypes related to the reference amplicons for the 500 resampled pooled subsets. Because of limitations on the amount of sequencing data we could generate, six replicates of amplicon 1 pool were sequenced and two replicates of amplicon 2 and 3 pools.

	Error rate for the 12.5% haplotype						Error rate for the 25% haplotype						Error rate for the 62.5% haplotype					
Amplicon	Individual	10x	30x	50x	100x	200x	Individual	20x	60x	100x	200x	400x	Individual	50x	150x	250x	500x	1000x
1	KA	.0135	.0104	.0073	.0096	.0093	KB	.0019	.0009	.0013	.0013	.0008	KC	.0011	.0009	.0013	.0013	.0008
1	KC	.0096	.0018	.0018	.0018	.0016	KA	.0008	.0000	.0000	.0000	.0013	KB	.0000	.0000	.0000	.0000	.0013
1	KB	.0125	.0047	.0029	.0028	.0029	KC	.0047	.0034	.0025	.0026	.0028	KA	.0001	.0003	.0002	.0013	.0006
1	KA	.0090	.0042	.0031	.0025	.0030	KC	.0039	.0031	.0024	.0019	.0029	KB	.0000	.0001	.0002	.0002	.0013
1	KC	.0068	.0025	.0019	.0019	.0022	KB	.0002	.0000	.0000	.0001	.0008	KA	.0000	.0000	.0000	.0000	.0006
1	KB	.0078	.0043	.0023	.0010	.0015	KA	.0013	.0008	.0008	.0004	.0013	KC	.0014	.0008	.0006	.0002	.0013
2	KA	.0164	.0154	.0035	.0021	.0019	KC	.0034	.0012	.0007	.0011	.0002	KB	.0000	.0004	.0000	.0007	.0000
2	KA	.0258	.0121	.0085	.0102	.0059	KB	.0008	.0001	.0000	.0001	.0000	KC	.0004	.0000	.0000	.0000	.0000
3	KC	.0400	.0107	.0066	.0018	.0049	KA	.0023	.0014	.0008	.0006	.0042	KB	.0002	.0002	.0002	.0002	.0002
3	KB	.0131	.0060	.0060	.0047	.0049	KC	.0115	.0005	.0006	.0000	.0000	KA	.0000	.0000	.0000	.0000	.0000
Mean		.0155	.0072	.0044	.0038	.0038		.0031	.0011	.0009	.0008	.0014		.0003	.0003	.0003	.0004	.0006

Scale: green = 0.00; yellow = 0.01; red = 0.05

**Table 11:** Error rates for the reconstructed haplotypes related to the reference amplicons for single-amplicon samples for the western grey individual KA.

	10x	20x	30x	50x	60x	100x	200x	400x	500x	1000x
Amplicon 1	.0010	.0000	.0000	.0000	.0000	.0000	.0000	.0000	.0000	.0000
Amplicon 2	.0032	.0000	.0000	.0000	.0000	.0000	.0000	.0000	.0000	.0000
Amplicon 3	.0115	.0033	.0011	.0000	.0000	.0000	.0000	.0000	.0000	.0000

Scale: green = 0.00; yellow = 0.01; red = 0.05

Low amount of errors in the reconstruction, error rate below 0.01, was obtained from [50x, 100x, 250x] sub-sampling for amplicon 1 and 3 and above [100x, 200x, 500x] for amplicon 2. Overall, the two Amplicon 3 experiments exhibited the highest amount of errors, with an average error rate of 0.0041 (Amplicon 1 average is 0.0023 and Amplicon 2 is 0.0037). In comparison, the error rate for the single-amplicon samples was nil for all amplicons when coverage was greater than 50x (Table 11).

### Sequencing experiment cost

In our experiment, three amplicons from three individuals were successfully sequenced and reconstructed using three barcoded samples. Typically, nine barcodes would have been required for a standard one-barcode-per-individual approach. In other words, the library preparation cost for our pooled approach is a third of the standard approach. To apply our method to larger numbers of individuals or amplicons, it is possible to combine it with standard barcoding. For example, multiple groups of three sequences sharing the same barcode can be sequenced. In each group, the sequences are pooled in different proportions (1:2:5). Therefore, when this. pooling approach is used, the cost of sequencing per individual is reduced as the number of samples increases per lane for the same number of barcodes. However, because higher coverage is required when pooling, the cost can increase as more lanes of sequencing are needed. Our pooling protocol may cost less for library preparation but cost more for sequencing. This is because for a target coverage *n* for the amplicon at the lowest concentration, we need to sequence two amplicons at *2n* and *5n*, respectively, to be able to separate the reads into the individual haplotypes.

We can estimate the total cost of this approach by considering a fixed cost per lane of a sequencing machine. Provided the number of reads produced per lane *n*_*r*_, the reads length *l*_*r*_, the fixed cost per lane *l*, the library preparation cost per sequenced sample *b*, the target amplicon/genome fragment length to sequence *g* and the required coverage *c*, the cost of both sequencing approaches can be calculated against an increasing number of individuals *i*. We assume that the individuals are grouped in pools of three. The pooling cost is calculated according to the following formula. Define $x=\left(\frac{i}{3}\right)*c$ as the minimum coverage required for one individual, and $y=\frac{\left(x+2y+5y\right)*g}{l_{r}*n_{r}}$ as the number of sequencing lane required to achieve the coverage of each sample with differential proportions. Then, the total cost of the experiment is equal to $\left(\frac{i}{3}\right)*b+y*l$.

**Fig 4:**
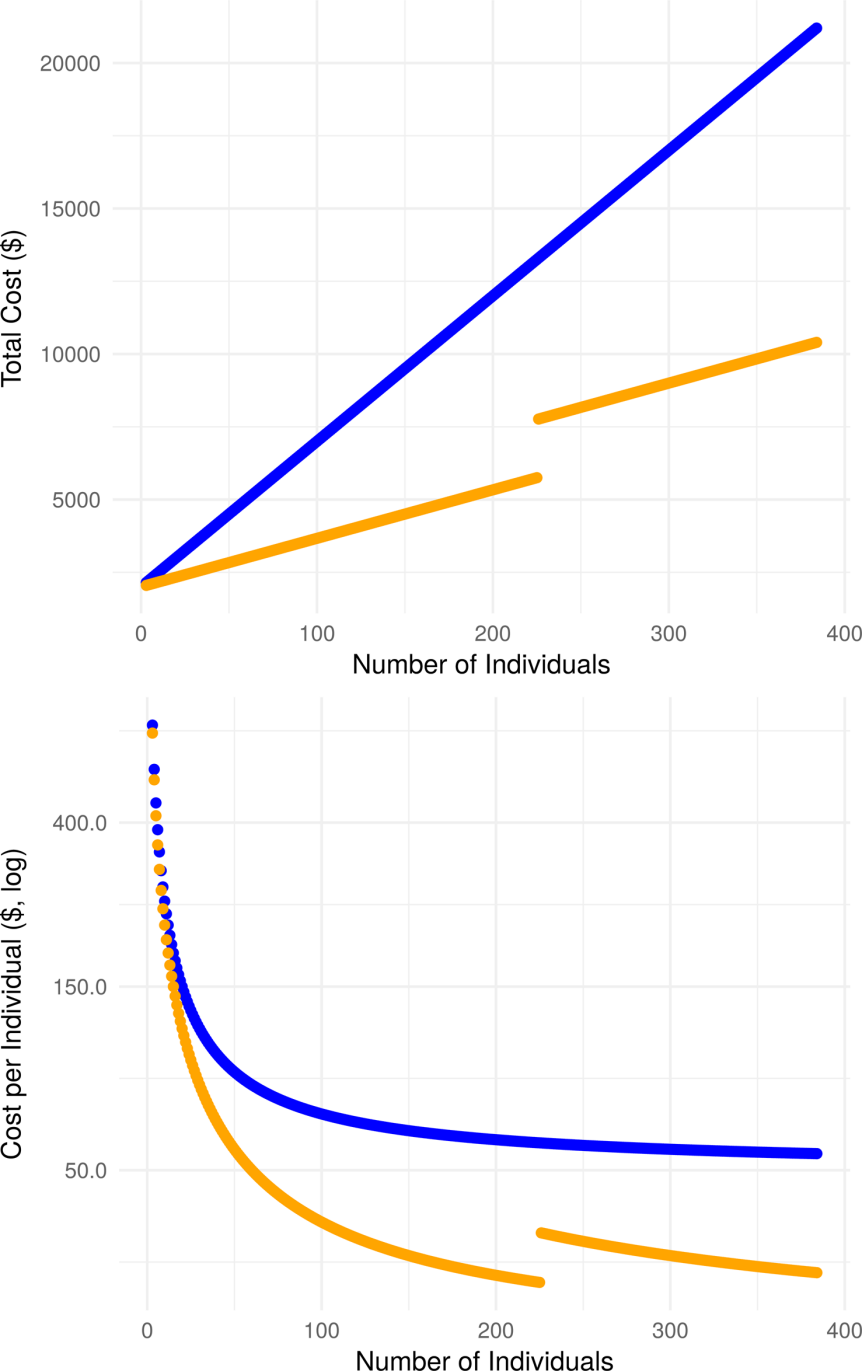
Sequencing cost calculator. A sequencing cost calculator is available to explore the total cost and the per individual cost of high-throughput experiments. In this example, the aim is to re-sequence an amplicon of 5,000 nucleotides. Sequencing output is set for an Illumina MiSeq machine; the number of reads is set to 20 million with read length of 150bp. A fixed cost per barcode is considered for the library preparation and the cost per sequencing lane is set to $2000, library preparation per individual is $50, target region size is 5,000bp at 1,000x coverage. Total cost increases with the number of individuals while per individual cost decreases. Line breaks correspond to the addition of a sequencing lane to achieve the requested coverage. See https://lmjr.shinyapps.io/application/ for different settings.

For example, using typical Illumina MiSeq System specifications (https://www.illumina.com/systems/sequencing-platforms/miseq/specifications.html) to sequence a 5,000 bp amplicon (Fig 4), the associated cost of applying our pooling protocol remains lower than for a standard approach where all individuals are uniquely barcoded, even though more lanes are required. Moreover, the difference in cost increases with the number of individuals added to the experiment; for 51 individuals (where individuals are pooled and sequenced in sets of three), the pooling approach is 37% cheaper than the barcoded approach and for 102 individuals, it reduces the cost to around 48%. The coverage per individual is equal for all individuals on the standard barcoded protocol but varies in the pooled approach. In the latter, we report coverage that corresponds to the individual at the lowest concentration (12.5%); coverage increases proportionally for the other two haplotypes at higher concentrations.

Because of the rapid improvement in the sequencing technologies and variation in the cost between different sequencing providers, we have developed a free online tool to calculate the cost of sequencing experiments. This tool can also evaluate the potential saving on cost that our pooling approach can provide (https://lmjr.shinyapps.io/application/). The tool allows users to vary the sequencing design and parameters (cost of a lane of sequencing, number of reads, read length, cost of library preparation per barcode, target genome/amplicon size and coverage) and display the cost of the experiment as a function of the total number of individuals to be sequenced.

## Discussion

Our results demonstrate that individual haplotypes can be reconstructed when pooled in a single sample by using different known amounts of DNA that are carefully chosen. Therefore, this strategy allows us to sequence a larger number of individuals at a reduced cost. In fact, our pooling approach can reduce the overall cost of a sequencing experiment up to a third depending on the sequencing settings. For now, our method can already be applied to pooled samples of three haploid amplicons.

The per-individual cost of the library preparation is a major factor to consider when deciding to use a pooling approach. If this cost is high then pooling individuals will significantly reduce the cost of the experiment. In the current experiments, pools of three individuals only were investigated. Pooling higher numbers of individuals would further reduce the cost of the library preparation. However, there is less distance between the expected proportions in the mixture. For example with pools of four, proportions 1:2:4:8 may be used to avoid ambiguities in the expected proportions. In this case, the resulting concentration of the haplotypes 1 to 3 when they are identical is 46.67% ((1+2+4)/15), which is close to the expected concentration of haplotype 4 (53%). To be able to discriminate successfully between all possible combinations, one would need even greater coverage depth, as evidenced in Table 10. For the case of diploid samples, our method could be applied to a set of 2 samples using input frequencies 25% and 75% (proportions 1:3). Although the number of haplotypes to reconstruct will increase from three to four, the number of frequency being less, the number of states in the HMM will be greatly reduced. A potential challenge would be to correctly phase each sample. We are presently developing protocols for larger numbers of haplotypes and diploid genomes which will greatly enhance the application scope of our method. Alternatively, it is possible to combine our pooling approach with standard barcoding, e.g. sequencing multiple pools of three amplicons, and greatly reduce the cost of experiments.

We used Bowtie 2 [20] to map the reads to the reference sequence. A limitation of this program is that reads are trimmed when part of the reads cannot be well aligned. As a result, the local coverage of the corresponding regions may be reduced in the final read alignment. This can also affect the proportion of reads from different haplotypes that are represented in the final alignment, because it is possible that only reads originating from some haplotypes are difficult to align and are subsequently discarded.

Since our method relies on recovering sequences using expected concentrations–and hence, coverage–as a marker for haplotype identity, factors that affect coverage, either across the genome or for individual haplotypes in the mixture, can influence the efficacy of our protocol and our algorithm.

First, a critical experimental step in our protocol is the accurate quantification of input DNA concentrations. In this study, we used a Qubit fluorometer which, according to the manufacturer, can measure DNA concentration within 1% of actual concentration when samples contain 10 ng/μL and up to 12% at lower concentrations. Departures from targeted proportions can result from pipetting errors when constructing the pools [25]. Automated liquid handling by a pipetting robot may be a helpful strategy for this purpose.

We have also identified factors that can affect the short-read coverage of the three haplotypes in our mixture. As previously reported [11–12], we found evidence that GC content affects the amount of coverage. While GC-rich regions can result in lower Illumina read coverage [26], our results showed positive correlations between coverage and GC content in the mitochondrial regions that were sequenced.

Coverage also varied within and between amplicons, as well as between the two species of kangaroo. The most difficult amplicon to reconstruct (amplicon 3) includes the hyper-variable mitochondrial control region. As noted above, the coverage for this region varied depending on whether the haplotype was most closely related to the eastern grey reference sequence. Western grey haplotype regions that are too divergent from the eastern grey reference will produce short reads that are difficult to map with standard mappers such as Bowtie, causing unexpected variations in the local proportions. High divergence between the western grey and eastern grey haplotypes in this region is therefore likely to explain differences in the coverage patterns which will then affect the performance of our algorithm.

Coverage also varied greatly within each amplicon. Local variation in coverage of one or two of the amplicons in the mixture can result in deviations from the expected proportions of the different haplotypes. Although the Dirichlet Multinomial distribution allowed us to take into account this variation, a method that can model the variation in coverage more accurately may potentially help haplotype reconstruction.

## Supporting information

S1 Appendix Hidden Markov Model for reconstructing pooled samples. Mathematical description of the algorithm, the coverage of consecutive windows is analysed to identify the most likely source of each identified nucleotide sub-sequence.
(PDF)

## References

[1] O’Neill EM, Schwartz R, Bullock CT, Williams JS, Shaffer HB, Aguilar-Miguel X, et al Parallel tagged amplicon sequencing reveals major lineages and phylogenetic structure in the North American tiger salamander (*Ambystoma tigrinum*) species complex. Molecular Ecology. 2013; 22(1): 111–129. doi: 10.1111/mec.12049 23062080 10.1111/mec.12049

[2] Mariac C, Scarcelli N, Pouzadou J, Barnaud A, Billot C, Faye A, et al Cost-effective enrichment hybridization capture of chloroplast genomes at deep multiplexing levels for population genetics and phylogeography studies. Molecular Ecology Resources. 2014; 14(6): 1103–1113. doi: 10.1111/1755-0998.12258 24690362 10.1111/1755-0998.12258

[3] Peñalba JV, Smith LL, Tonione MA, Sass C, Hykin SM, Skipwith PL, et al Sequence capture using PCR-generated probes: a cost-effective method of targeted high-throughput sequencing for nonmodel organisms. Molecular Ecology Resources. 2014; 14(5): 1000–1010. doi: 10.1111/1755-0998.12249 24618181 10.1111/1755-0998.12249

[4] Quick J, Grubaugh ND, Pullan ST, Claro IM, Smith AD, Gangavarapu K, et al Multiplex PCR method for MinION and Illumina sequencing of Zika and other virus genomes directly from clinical samples. Nature Protocols. 2017; 12(6): 1261–1276. doi: 10.1038/nprot.2017.066 28538739 10.1038/nprot.2017.066 PMC5902022

[5] Leguia M, Cruz CD, Felices V, Torre A, Troncos G, Espejo V, et al Full-genome amplification and sequencing of Zika viruses using a targeted amplification approach. Journal of Virological Methods. 2017; 248: 77–82. doi: 10.1016/j.jviromet.2017.06.005 28633961 10.1016/j.jviromet.2017.06.005

[6] Schlötterer C, Tobler R, Kofler R, Nolte V. Sequencing pools of individuals—mining genome-wide polymorphism data without big funding. Nature Reviews Genetics. 2014; 15(11): 749–763. doi: 10.1038/nrg3803 25246196 10.1038/nrg3803

[7] Gautier M, Foucaud J, Gharbi K, Cézard T, Galan M, Loiseau A, et al Estimation of population allele frequencies from next-generation sequencing data: pool-versus individual-based genotyping. Molecular Ecology. 2013; 22: 3766–3779. doi: 10.1111/mec.12360 23730833 10.1111/mec.12360

[8] Futschik A, Schlo C. The Next Generation of Molecular Markers From Massively Parallel Sequencing of Pooled DNA Samples. 2010; 218:207–218. http://doi.org/10.1534/genetics.110.114397 10.1534/genetics.110.114397 PMC2940288 20457880

[9] Ferretti L, Ramos-Onsins SE, Pérez-Enciso M. Population genomics from pool sequencing. Molecular Ecology. 2013 http://doi.org/10.1111/mec.12522 10.1111/mec.12522 24102736

[10] McComish BJ, Hills SFK, Biggs PJ, Penny D. Index-free de novo assembly and deconvolution of mixed mitochondrial genomes. Genome Biology and Evolution. 2010; 2:410–24. doi: 10.1093/gbe/evq029 20624744 10.1093/gbe/evq029 PMC2997550

[11] Aird D, Ross MG, Chen WS, Danielsson M, Fennell T, Russ C, et al Analyzing and minimizing PCR amplification bias in Illumina sequencing libraries. Genome Biology. 2011; 12(2), R18 doi: 10.1186/gb-2011-12-2-r18 21338519 10.1186/gb-2011-12-2-r18 PMC3188800

[12] Minoche AE, Dohm JC, Himmelbauer H. Evaluation of genomic high-throughput sequencing data generated on Illumina HiSeq and Genome Analyzer systems. Genome Biology. 2011; 12(11): R112 doi: 10.1186/gb-2011-12-11-r112 22067484 10.1186/gb-2011-12-11-r112 PMC3334598

[13] Ekblom R, Smeds L, Ellegren H. Patterns of sequencing coverage bias revealed by ultra-deep sequencing of vertebrate mitochondria. BMC Genomics. 2014; 15, 467 doi: 10.1186/1471-2164-15-467 24923674 10.1186/1471-2164-15-467 PMC4070552

[14] Van den Hoecke S, Verhelst J, Saelens X. Illumina MiSeq sequencing disfavours a sequence motif in the GFP reporter gene. Scientific Reports. 2016; 6: 26314 doi: 10.1038/srep26314 27193250 10.1038/srep26314 PMC4872057

[15] State of New South Wales and Office of Environment and Heritage. New South Wales Commercial Kangaroo Harvest Management Plan 2017–21, 2016 Annual Report. 2017.

[16] Nilsson MA, Gullberg A, Spotorno AE, Arnason U, Janke A. Radiation of Extant Marsupials after the K/T Boundary: Evidence from Complete Mitochondrial Genomes. Journal of Molecular Evolution. 2003; 57(SUPPL. 1): 3–12. 10.1007/s00239-003-0001-8 15008398

[17] Dodt WG, McComish BJ, Nilsson MA, Gibb GC, Penny D, Phillips MJ. The complete mitochondrial genome of the eastern grey kangaroo (Macropus giganteus). Mitochondrial DNA. 2016; 27(2), 1366–1367. doi: 10.3109/19401736.2014.947583 25103427 10.3109/19401736.2014.947583

[18] Kearse M, Moir R, Wilson A, Stones-Havas S, Cheung M, Sturrock S, et al Geneious Basic: an integrated and extendable desktop software platform for the organization and analysis of sequence data. Bioinformatics. 2012; 28(12): 1647–1649. doi: 10.1093/bioinformatics/bts199 22543367 10.1093/bioinformatics/bts199 PMC3371832

[19] MATLAB and Bioinformatics Toolbox Release 2016b, The MathWorks, Inc., Natick, Massachusetts, United States.

[20] Langmead B, Salzberg S. Fast gapped-read alignment with Bowtie 2. Nature Methods. 2012; 9: 357–359. doi: 10.1038/nmeth.1923 22388286 10.1038/nmeth.1923 PMC3322381

[21] Garrison E, Marth G. Haplotype-based variant detection from short-read sequencing. arXiv preprint arXiv:1207.3907 [q-bio.GN] 2012.

[22] Wu SH, Schwartz RS, Winter DJ, Conrad DF, Cartwright RA. Estimating error models for whole genome sequencing using mixtures of Dirichlet-multinomial distributions. Bioinformatics. 2017; 266(15): 554–71. 10.1093/bioinformatics/btx133 PMC5860108 28334373

[23] Zhou H, Zhang Z. Matlab MGLM Toolbox Version 1.0.0. 2017; Available online.

[24] Viterbi A. Error bounds for convolutional codes and an asymptotically optimum decoding algorithm. IEEE transactions on Information Theory. 1967; 13(2): 260–269.

[25] Sham P, Bader JS, Craig I, O’Donovan M, Owen M. DNA Pooling: a tool for large-scale association studies. Nature Reviews Genetics. 2002; 3(11): 862–871. doi: 10.1038/nrg930 12415316 10.1038/nrg930

[26] Chen YC, Liu T, Yu CH, Chiang TY, Hwang CC. Effects of GC Bias in Next-Generation-Sequencing Data on De Novo Genome Assembly. PLoS ONE. 2013; 8(4), e62856 doi: 10.1371/journal.pone.0062856 23638157 10.1371/journal.pone.0062856 PMC3639258

