# Supplementary material for: Reassembling haplotypes in a mixture of pooled amplicons when the relative concentrations are known: A proof-of-concept study on the efficient design of next-generation sequencing strategies
Source: PLoS One. 2018 Apr 5;13(4):e0195090. doi: 10.1371/journal.pone.0195090 (PMC5886459; doi:10.1371/journal.pone.0195090)
Supplement: S1 Appendix — Mathematical description of the algorithm, the coverage of consecutive windows is analysed to identify the most likely source of each identified nucleotide sub-sequence. (PDF) [file pone.0195090.s001.pdf]

Appendix: Hidden Markov model for reconstructing pooled haplotypes

Let's consider an analysis window w_t of length l starting at position t in the multiple sequence alignment of length T built from the mapping of the reads to the reference. For each of the consecutive windows positioned at $t_1, ..., t_T$ in the alignment, only the most frequent, i.e. highest read coverage, nucleotide subsequences are retained and noted $\mathbf{o}_t = (o_{t,1}, ..., o_{t,x})$ where $x <= 3$ (Figure 1, ①).

Let $h_{t,n}$ denote the nucleotide sequence for the haplotype n in w_t for $n = 1, 2, 3$. Let $q = (q_1, ..., q_T)$ the hidden states of the HMM mapping $\mathbf{o}_t$ to the haplotype nucleotide sequences $\mathbf{h}_t = (h_{t,1}, h_{t,2}, h_{t,3})$ being reconstructed (Figure 1, ③). The states represent the combinations $\binom{3}{3}$ with replacement of $\mathbf{o}_t$ to $\mathbf{h}_t$ (Table 1).

Table 1: The 27 possible states of the HMM correspond to the different combinations of three haplotypes.

	$h_{t,1}$	$h_{t,2}$	$h_{t,3}$
s_1	$o_{t,1}$	$o_{t,1}$	$o_{t,1}$
s_2	$o_{t,1}$	$o_{t,1}$	$o_{t,2}$
s_3	$o_{t,1}$	$o_{t,1}$	$o_{t,3}$
s_4	$o_{t,1}$	$o_{t,2}$	$o_{t,1}$
s_5	$o_{t,1}$	$o_{t,2}$	$o_{t,2}$
s_6	$o_{t,1}$	$o_{t,2}$	$o_{t,3}$
...	...		
s_{27}	$o_{t,3}$	$o_{t,3}$	$o_{t,3}$

Let the initial state probability be noted $\pi = (\pi_1, ..., \pi_{27})$, where $\pi_i = P(q_1 = s_i)$, where s_i is the ith state for $i = 1, ..., 27$. The transition probability from state s_i at position t to state s_j at position $t+1$ is noted $a_{ij}(t) = P(q_{t+1} = s_j | q_t = s_i)$ where $t \in \{t_1, ..., t_T\}$ and $j = 1, ..., 27$. The states transition probability matrix at w_t is A_t, $A = \{A_1, ..., A_T\}$.

$$a_{ij}(t) = \sum_{n=1}^{3} d(h_{t,n}(2, l), h_{t+1,n}(1, l-1)),$$

where d is the Hamming distance. The emission probability at state s_i is $b_i(\mathbf{o}_t) = P(\mathbf{o}_t | q_t = s_i, \theta_t) \sim DM(\theta_t)$, where DM denotes the Dirichlet-multinomial distribution. The parameter θ_t is the local count of the x most frequent subsequences of w_t. The joint distribution of the HMM is therefore

$$P(O_t, q | \theta_t, A, \pi) = \pi_1 b_1(\mathbf{o}_1) \prod_{t=2}^{T} a_{ij}(t) b_j(\mathbf{o}_t).$$

Given a sequence of observations O_t up to position t, the haplotypes are reconstructed by finding the best sequence of HMM states. Let $\delta_i(t)$ represent the maximal value of the joint likelihoods of observation sequence and associated HMM state sequence while in state s_i.

$$\delta_i(t) = \max_{i=1,...,27} P(O_t, q_{t-1}, q_t = s_i).$$

The optimal sequence can be computed by calculating the partial likelihood as a recursion

$$\delta_j(t+1) = \max_i \delta_i(t) a_{ij} b_j(\mathbf{o}_{t+1}),$$

for all states j.

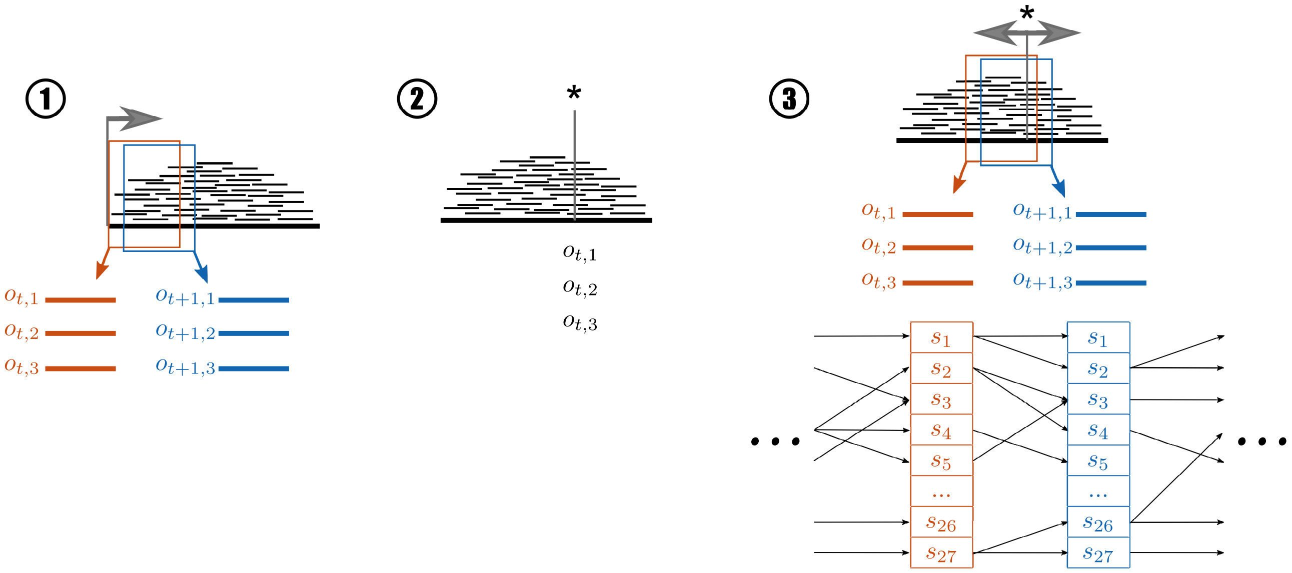

Figure 1: HMM allows to measure the read coverage of consecutive analysis windows and to identify the most likely source, i.e. haplotype, of each identified nucleotide sub-sequence.

To find the starting point of the HMM in the alignment, multinomial distributions with parameters $\binom{\lambda}{3}$ with $\lambda = (0.125, 0.25, 0.625)$ the set of known proportions for all w_t. The HMM then starts at the window with maximum likelihood for state s_6, where all haplotypes are different, offering the best estimate of their local proportions (Figure 1, ②).
